# Global analysis of phase locking in gene expression during cell cycle: the potential in network modeling

**DOI:** 10.1186/1752-0509-4-167

**Published:** 2010-12-03

**Authors:** Shouguo Gao, John L Hartman IV, Justin L Carter, Martin J Hessner, Xujing Wang

**Affiliations:** 1 Department of Physics, the University of Alabama at Birmingham, Birmingham, Alabama, 35294, USA; 2 The Comprehensive Diabetes Center, the University of Alabama at Birmingham, Birmingham, Alabama, 35294, USA; 3 Department of Genetics, the University of Alabama at Birmingham, Birmingham, Alabama, 35294, USA; 4 The Max McGee National Research Center for Juvenile Diabetes, Department of Pediatrics at the Medical College of Wisconsin and the Children's Research Institute of the Children's Hospital of Wisconsin, 8701 Watertown Plank Road, Milwaukee, Wisconsin, 53226, USA; 5 The Human and Molecular Genetics Center, The Medical College of Wisconsin, 8701 Watertown Plank Road, Milwaukee, Wisconsin, 53226, USA

## Abstract

**Background:**

In nonlinear dynamic systems, synchrony through oscillation and frequency modulation is a general control strategy to coordinate multiple modules in response to external signals. Conversely, the synchrony information can be utilized to infer interaction. Increasing evidence suggests that frequency modulation is also common in transcription regulation.

**Results:**

In this study, we investigate the potential of phase locking analysis, a technique to study the synchrony patterns, in the transcription network modeling of time course gene expression data. Using the yeast cell cycle data, we show that significant phase locking exists between transcription factors and their targets, between gene pairs with prior evidence of physical or genetic interactions, and among cell cycle genes. When compared with simple correlation we found that the phase locking metric can identify gene pairs that interact with each other more efficiently. In addition, it can automatically address issues of arbitrary time lags or different dynamic time scales in different genes, without the need for alignment. Interestingly, many of the phase locked gene pairs exhibit higher order than 1:1 locking, and significant phase lags with respect to each other. Based on these findings we propose a new phase locking metric for network reconstruction using time course gene expression data. We show that it is efficient at identifying network modules of focused biological themes that are important to cell cycle regulation.

**Conclusions:**

Our result demonstrates the potential of phase locking analysis in transcription network modeling. It also suggests the importance of understanding the dynamics underlying the gene expression patterns.

## Background

A major goal of systems biology is to integrate biological functions of individual genes in terms of their interactions. Time course gene expression profiling, which can capture the global transcriptional responses to signals during a biological process of interest, offers a major data source to achieve this goal [1].

In network modeling of gene expression data, assessing pair-wise relationships is often a starting point. In early days, correlation coefficient [2,3], Euclidean distance, as well as their variations, such as partial correlations, empirical Bayes and bootstrap methods [4], were used. They are effective for computing direction free linear dependence when the data are independent. Networks constructed this way are essentially co-expression networks. While having the appeal of being simple and intuitive, correlation metrics have limitations when applied to time course data. They assume independence of the order of the data points, while in reality the data at each time step depend on the previous time points. Ignoring the inter-time point dependence not only loses sensitivity toward detecting interactions but could also lead to erroneous predictions [5].

Significant phase shift in the timing of expression changes have also been observed for highly associated genes [6]. Some studies tried to identify the phase lag directly by shifting gene expression time series with respect to each other until the optimal alignment is reached. For instance, Qian *et al *proposed a local clustering approach based on optimal pair-wise alignment through dynamic programming [7]; Schmitt *et al *used the Pearson's correlation [8], Balasubramaniyan *et al *used the Spearman rank correlation [9], Pereda *et al *used cross correlation [10], to compute the maximum time-lagged similarity between two transcript profiles, and utilized the results to identify clusters. The degree of lag varies widely in different gene pairs, and these approaches need multiple runs to find the lag that best aligns each pair. The performance of the alignment depends on whether the lags are close to integer numbers of the sampling steps of the experiment.

More sophisticated methods were also developed. Aach and Church implemented both simple and interpolative time warping based on dynamic programming to identify an optimal alignment of two gene expression time series [11]. Expanding this approach, Liu and Müller proposed a non-parametric time-synchronized iterative mean updating technique to construct modes of temporal structure in gene expression profiles [12]. Bar-Joseph *et al. *[13,14] developed an approach to align temporal data sets using piecewise spline fitting, extracting shift and stretch parameters for each data set. Butte *et al. *[6] utilized digital signal-processing tools, including power spectral densities, coherence, transfer gain, and phase shift, to find pair-wise gene associations based on periodically expressed time-invariant gene profiles. More recently, a hidden Markov model based approach was utilized to infer the timing in gene expression changes under different experimental conditions [15].

Linear and non-linear multivariate analysis and signal processing techniques were also introduced to analyze time series microarray data [16]. Several studies used pair wise mutual information to infer interactions and regulatory relationships between genes [17,18]. This method assumes a fixed time delay, which might not be true across different experimental conditions. In frequency domain time series analysis, causality and interrelationship among the components can be studied using coherence and partial coherence. Graphical models based on such analysis have been studied by Butte *et al *[6] and Salvador *et al *[19]. However, Albo *et al *[20] showed that partial coherence-based causality measures are sensitive to measurement noise.

Apparently, more studies are needed to fully utilize the dynamics underlying the temporal gene expression pattern, and to better understand the complex spatial-temporal architecture of transcriptome. Recently, increasing evidence, including those from the advancement of single-cell time course gene expression profiling technologies [21], suggest that like other complex dynamic systems in nature, synchrony through oscillation and frequency modulation is a general strategy for an organism to coordinate the transcription of multiple target genes in responses to external signals [22-26]. Examples include the p53-Mdm2 feedback loop [24,25], the NF-κB signaling pathway [27], and calcium responsive pathways [23]. These further emphasize the need of new methods to study and utilize the dynamics. The oscillations in gene expression, like other oscillations in biological systems [28], are most often pulsatile or relaxed oscillations rather than harmonic, thus calling for mathematical methods rooted from phase space analysis [29,30].

In this study, we investigate the potential of network inference using the phase locking analysis technique [31]. This approach is based on the following concepts originating from nonlinear dynamics [29,30]: if two time series interact with each other, there will be a process of rhythmic adjustment resulting from the interaction, leading to phase locking. Phase-locked oscillators progress through their trajectories in phase space at the same pace (1:1 locking), or rational ratios with respect to each other (*m*:*n *locking, *m *and *n *being integers). Conversely, such phase locking phenomenon can be utilized to infer interaction between two dynamic systems, even for weak interactions [31]. Recently Kim *et al *clustered genes of synchronized oscillatory pattern (1:1 phase locking) during yeast cell cycle, and observed that genes in the same cluster were closely associated, as evidenced by the sharing of GO terms and BioGRID interactions [32]. In this study we will apply the phase locking analysis to the Stanford yeast cell cycle data [33,34], and examine the phase locking (including higher order locking) between transcription factors and targets, between gene pairs with prior evidence of other types of interaction, and between cell cycle genes. Based on the results, we will propose a new network inference approach utilizing the phase locking index, and examine the modular structure of the networks constructed and the biological themes shared by genes in the network modules.

## Results

### Phase locking of interacting genes

#### Distribution of λ

**Figure 1:**
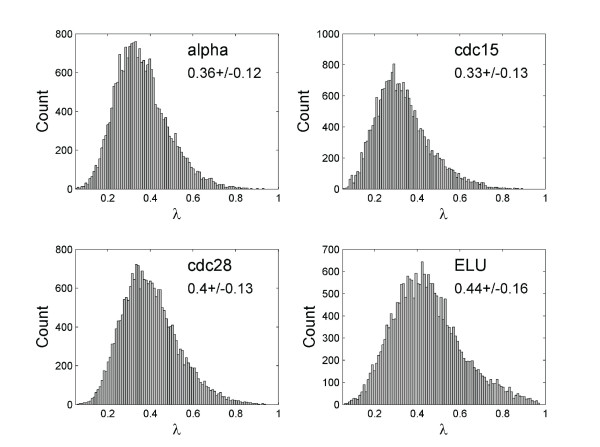
**Distribution of the phase locking index λ**.

We first examined the distribution of phase locking index *λ *in all four datasets of he Stanford yeast cell cycle study [33,34], the histograms are given in Figure 1. In each case the distribution is close to normal (p < 1e-10, KS test), indicating that the way we define the threshold *λ *for significant locking, 2 standard deviations above mean, is reasonable. In all histograms there is a hint of a high-*λ *tail, likely contributed from gene pairs that are phase locked.

#### Phase locking between the cell cycle regulating TFs and their targets

Following the original gene expression study of cell cycles [33,34], several groups have investigated yeast transcription binding using the ChIP-chip technology [35,36]. These data provide useful information of which genes are potentially transcription regulation targets of each TF. We have obtained the data from Simon *et al*, where the promoter binding by the 9 known cell cycle regulating TFs were studied [36]. Both ChIP-chip and microarray data are noisy, and we found no direct quantitative dependence of *λ *or *r *on the binding p-value (*r *< 0.1).

**Figure 2:**
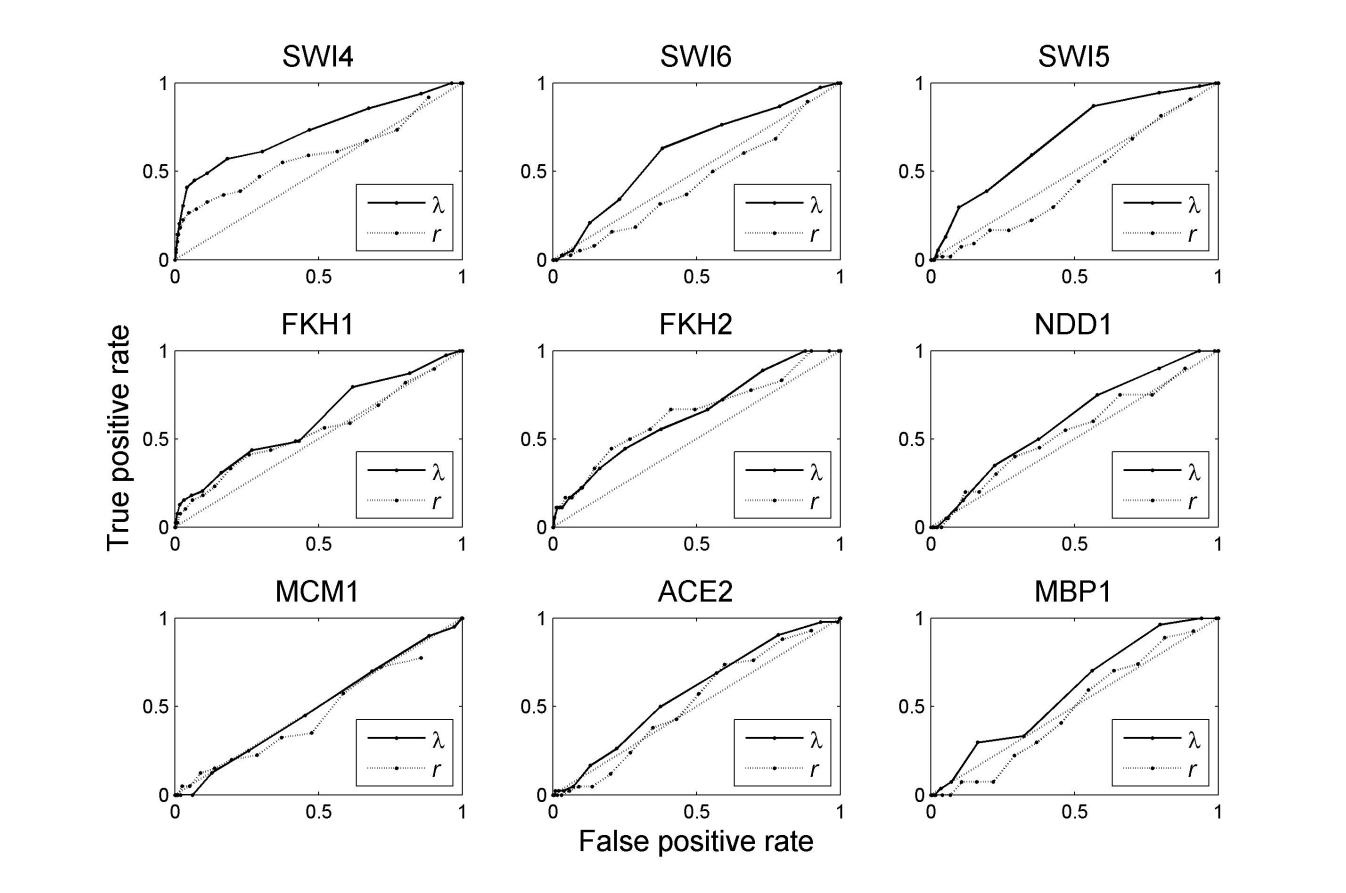
**The efficiency of using *r *and *λ *to distinguish targets from non-targets of transcription factors**. Overall, *λ *is significantly better than either *r *(p < 1e-9) or a random classifier (p < 1e-3). Plotted are data from the alpha factor dataset.

**Figure 3:**
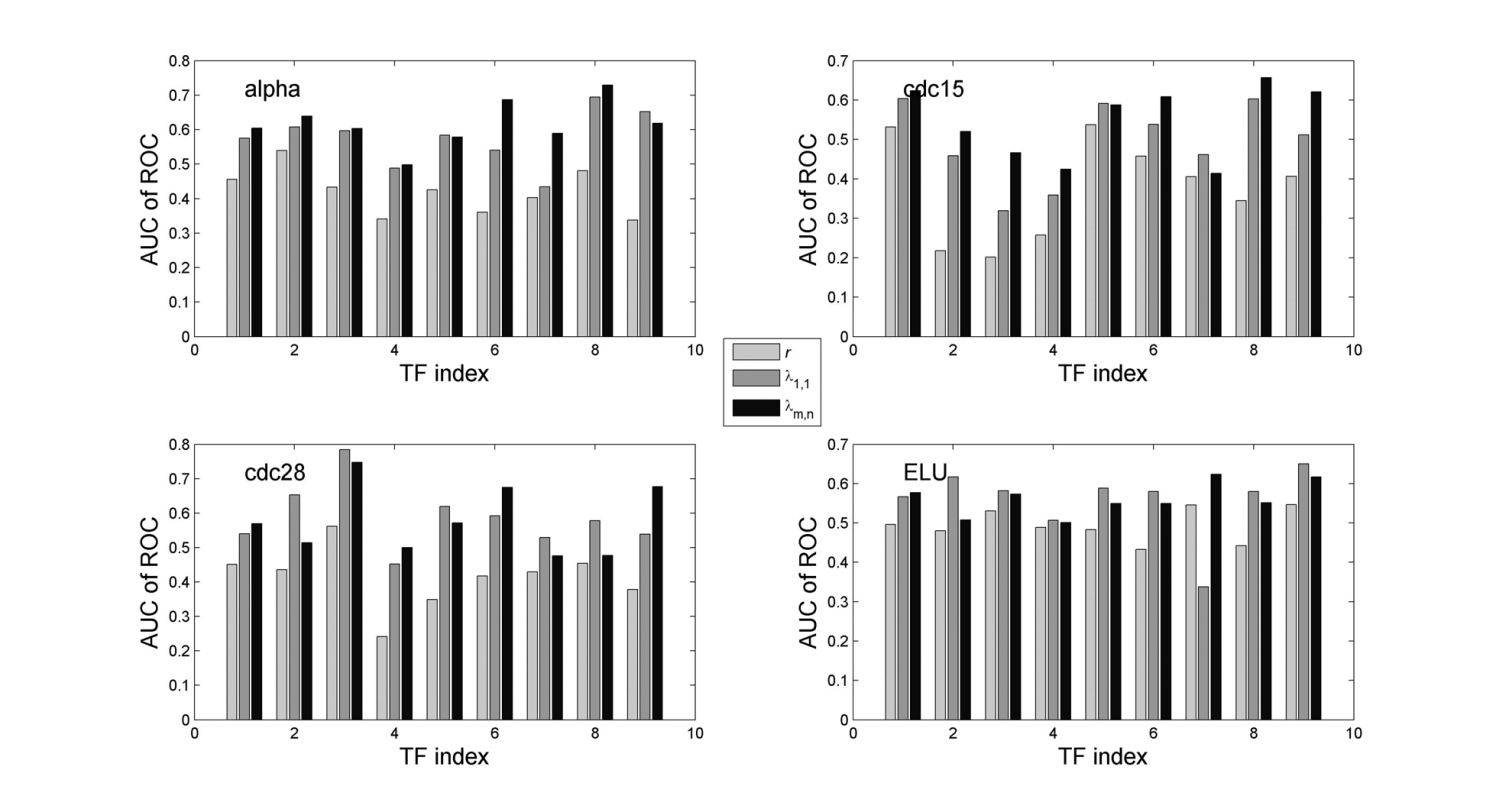
**AUC of ROC of the nine TFs**. Including the consideration of higher order locking significantly improved the performance.

However, we obtained interesting results when using the target and non-target control groups as benchmarks. The receiver operating characteristic (ROC) curve was used to determine whether or not *λ *can potentially discriminate targets from non-targets. ROC is a graphical plot of the sensitivity versus 1-specificity, namely the fraction of true positives versus the fraction of false positives, as the discrimination threshold of a classifier is varied. The area under curve (AUC) reflects the performance. The ROC of a random classifier would be a 45° line with AUC = 0.5. In Figure 2 the ROC plot for each TF in alpha factor arrest experiment is given. Overall, *λ *is significantly better than either a random classifier or *r *(p < 0.001 in both cases), suggesting that expression levels from TF and target genes are likely to exhibit phase locking. In contrast, the expression levels of TF and their targets seem to correlate little. In fact, using *r *does not fare any better than a random classifier (p > 0.5, t-test). The results from other datasets are similar, and the AUC of ROC plots from all four datasets are summarized in Figure 3. The list of target genes that exhibit significant phase locking with the TF is given in Additional file 1.

#### Phase locking of BioGRID gene pairs

To further investigate the potential of phase locking analysis in network inference, we examined phase locking between gene pairs that have evidence of other types of interaction according to BioGRID http://www.thebiogrid.org. BioGRID is a freely accessible database of physical (protein-protein) and genetic interactions, curated from high-throughput data and literature [37,38]. Of all possible gene pairs in our data sets, ~53,000 are annotated in BioGRID. We constructed five Bootstrapping [39] sets that consisted of the same number of BioGRID gene pairs, randomly sampled from all possible pairs. The distribution of phase locking index was examined in each group. We found that the distribution for the BioGRID pairs is skewed toward higher λ than the Bootstrapping sets (p < 1e-5, KS test).

**Figure 4:**
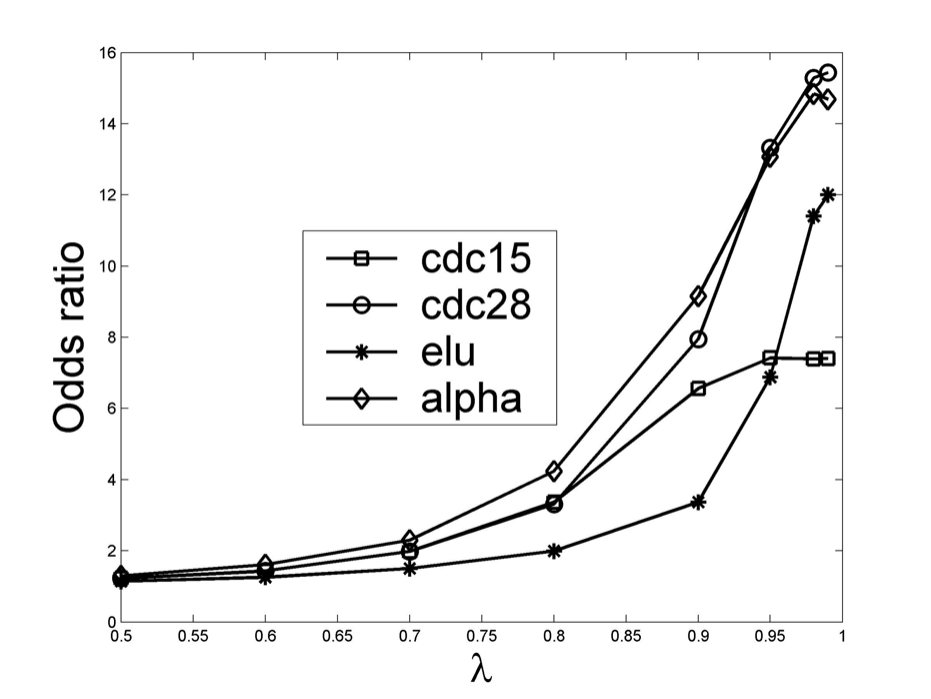
**Significantly enhanced presence of phase locking in BioGRID gene pairs**.

The odds ratio (OR) of the enrichment of phase locked pairs at a given threshold value *λ *_0_, when there is prior evidence of interaction according to BioGRID, can be calculated by:

(11) $$\text{Odds Ratio}=\frac{P(\lambda >\lambda_{0}|\text{BioGRID})}{P(\lambda >\lambda_{0}|\text{Bootstrapping})}$$

where *P*(*λ *>*λ *_0_|BioGRID) is the probability of a BioGRID gene pair having a significant phase locking *λ *>*λ *_0 _, and *P*(*λ *>*λ *_0_|Bootstrapping) is such probability of a Bootstrapping gene pair. The OR of the four datasets at different *λ *_0 _is given in Figure 4. Evidently, it increases with more stringent *λ *_0_, and is significantly greater than 1 above the cutoff values of *λ *that we used in this study (~0.75). These results further validate the idea that *λ *can be utilized to identify interacting gene pairs.

#### Phase locking among cell cycle genes

**Figure 5:**
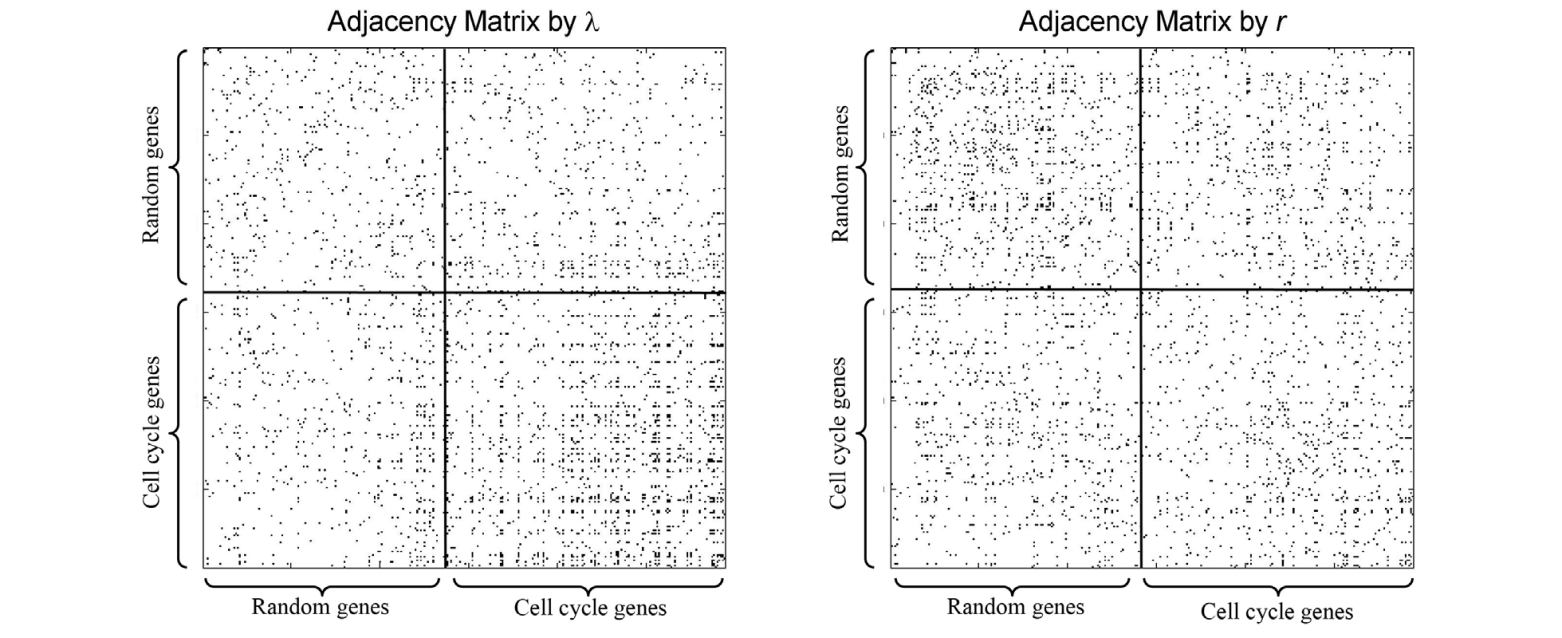
**The cell cycle genes form a densely connected subnetwork according phase locking**. Presented here are 2 D colormaps of the adjacency matrix *A *as defined by the phase locking index *λ *and the correlation coefficient *r*. Pixels are represented by black color if *A_i, j _*= 1, white if *A_i, j _*= 0. The diagonal elements *A_i, j _*were set to zero to avoid obscuring the interaction pattern between different genes.

**Figure 6:**
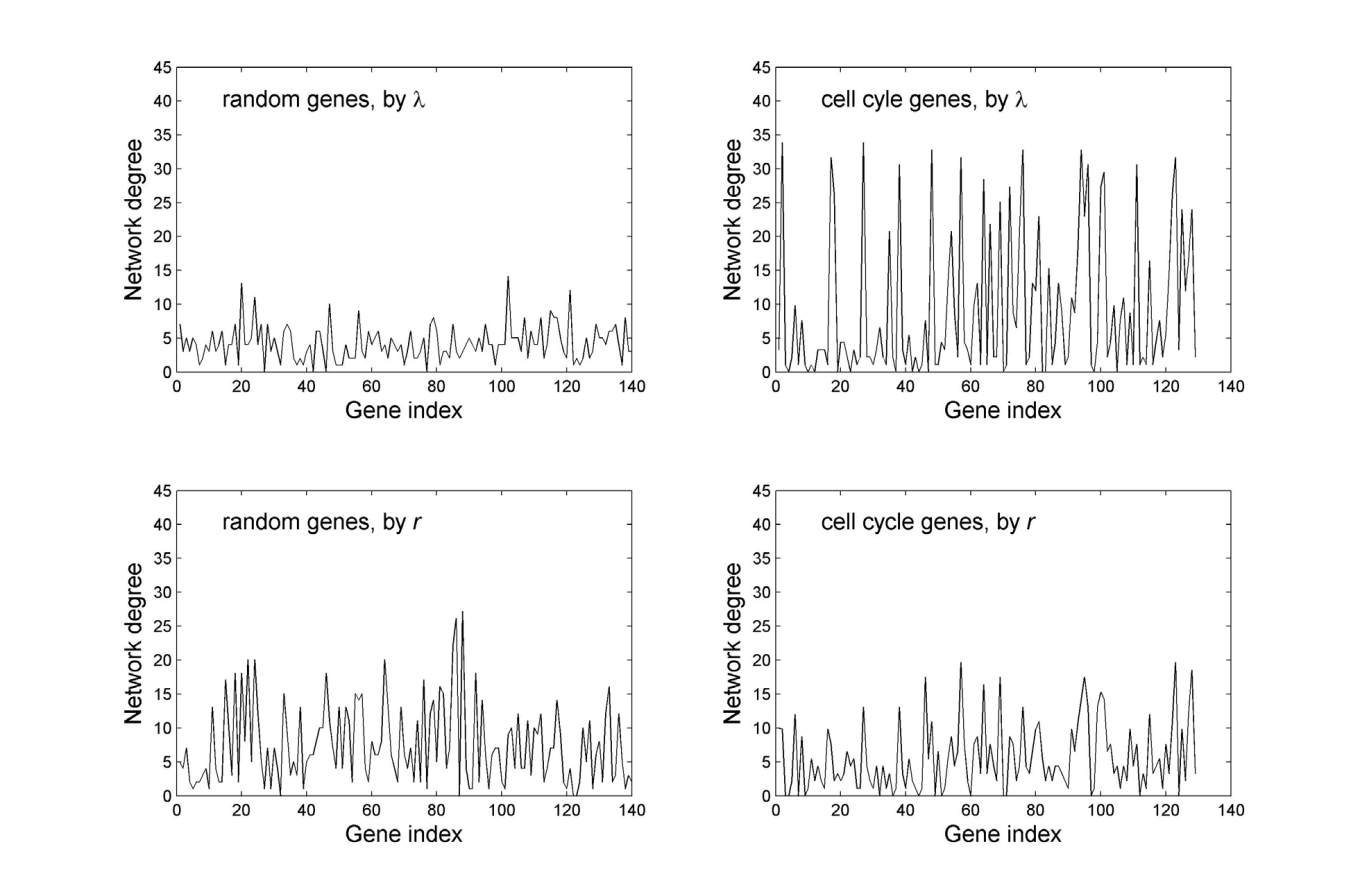
**Comparison of network degrees in the networks of random genes and cell cycle genes**. According to λ, the cell cycle genes clearly form a highly connected subnetwork, while random genes are sparsely connected. In contract, the two groups of genes show no connectivity difference according to *r*. Data presented are from the alpha factor synchronization dataset.

We anticipate the expression of the 144 cell cycle genes to show significant coordination, as they should all respond to the master signals that drive the cell cycle, and form an interaction network that together regulate the cell cycle progression. We found that indeed they exhibit phase locking among themselves more often than with random genes or than within random genes. On the other hand, when evaluated by the correlation coefficient *r *of their expression levels, cell cycle genes are not different from random genes. To illustrate the point we present in Figure 5 colormaps of the adjacency matrix A from the alpha factor arrest dataset, where the non-zero elements are presented by a black pixel. According to λ, in the subregion of cell cycle genes, there are significantly more black pixels than in the subregion of random genes, or in the subregion of cross interactions. To give a more quantitative description in Figure 6 we give the network degrees of all genes in the network. Evidently, based on phase locking, the cell cycle genes form a highly connected subnetwork, whilst random genes are sparsely connected. In sharp contrast, according to *r*, there is literally no difference between the cell cycle genes and the random genes, both with moderate connectivity. Results from the other three data sets are similar. Note that in each case the total number of genes is less than their purported values (144 and 150), this is because we have removed genes with more than one missing values in their time series measurements.

#### Phase lags and m:n phase locking

**Figure 7:**
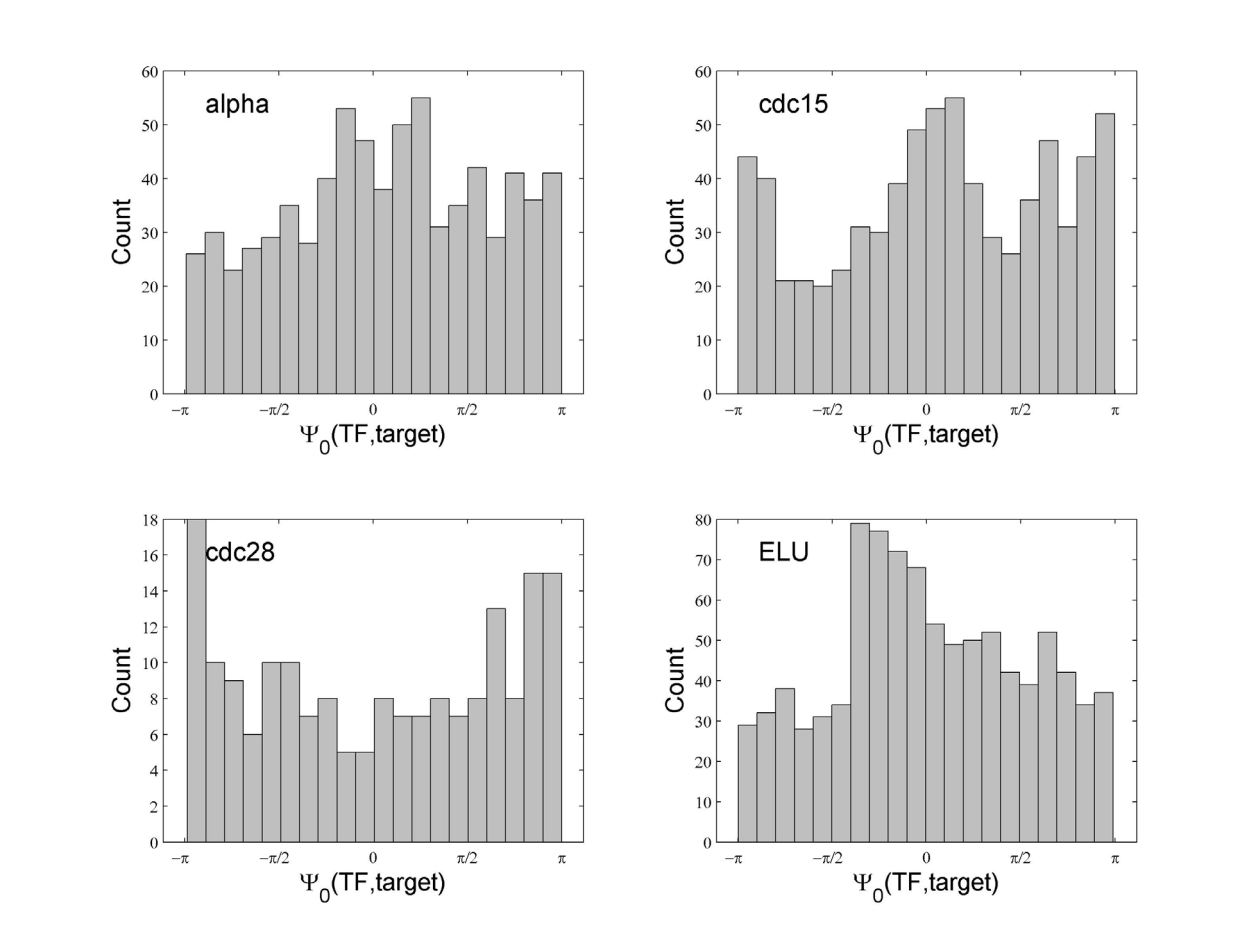
**Distribution of phase lags between TF and phase locked targets**.

In general we found that the phase lag Ψ_0 _between locked pairs varies widely, and most of them are not close to 0. This is consistent with observations made by others [6,40]. Figure 7 gives the distribution of the relative phase Ψ_0 _as defined in equation 7 of phase locked TF and target pairs. They span the whole range of [-π, π], with no consistent pattern. This is not surprising as it is the protein of the TF that interact directly with the target gene transcription, not the expression of the TF transcript [7,41]. Indeed, it has been observed that response delay varies widely in gene expression regulation [40]. Additional file 2 presents some examples of phase locked BioGRID gene pairs with significant phase lags, where the correlations are low, even with time-lagged correlations. More examples and movies of phase lagged gene pairs are available at our website http://zen.dom.uab.edu:8080/phase/demo/. The universal existence of phase lags and their variation suggest the advantage of a method like the phase locking analysis that can automatically account for them. The pair-wise alignment approaches will need to align each pair individually thus adding an extra step in network modeling.

**Figure 8:**
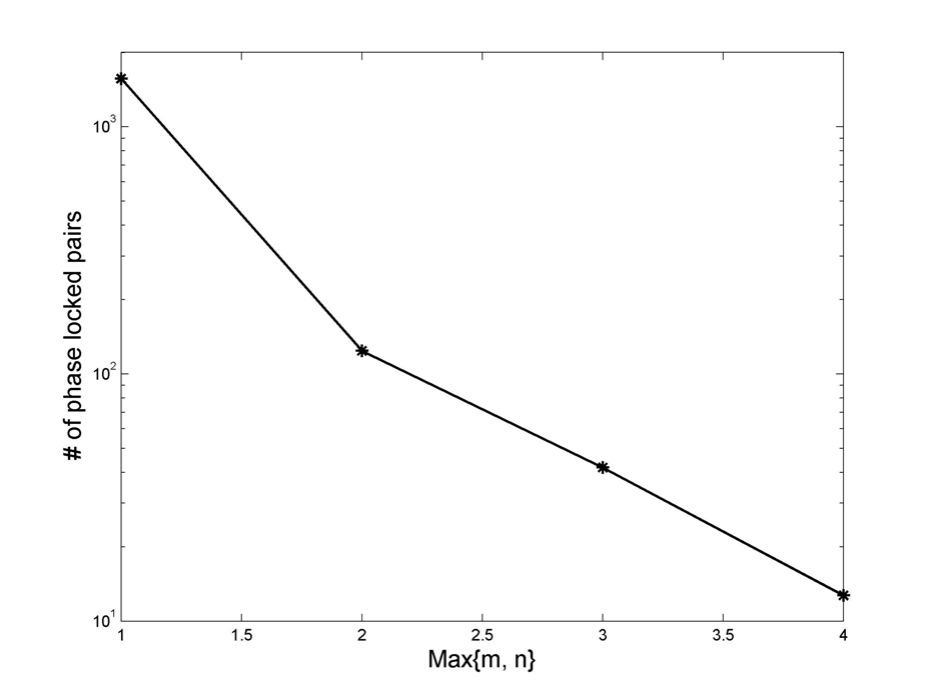
**Higher order phase locking in gene expression changes**. The number of phase locked gene pairs decrease exponentially with max{m, n}.

In this study we included the consideration of *m:n *phase locking, up to Max{*m*, *n*} = 4. We found that there were a significant number of gene pairs exhibiting higher than 1:1 locking. The number breakdowns are listed in Additional file 3. In the cell cycle gene analysis, on average there are 612.5 gene pairs in each data set that exhibit significant phase locking. Out of these pairs, ~5.7% are from higher order locking, with ~half contributed from the 1:2 and 2:1 locking. In the TF-target pairs, the proportion of higher order locking is significantly higher at ~10%. On average, in each dataset there are 1747 significantly phase locked TF-target pairs. 178 are due to higher than 1:1 locking, and ~70% of them are contributed from the 1:2 or 2:1 locking. The number of phase locked pairs, versus Max{*m*, *n*}, is given in Figure 8. Interestingly, an exponential dependence is evident. Including higher order phase locking significantly improved the performance of *λ *as a classifier to detect TF targets. In Figure 3 we also included results that only considered 1:1 locking. Clearly, the performance is not as good as when higher order locking is included (p ~ 0.02), though it is still better than *r *or a random classifier (p < 0.05).

**Figure 9:**
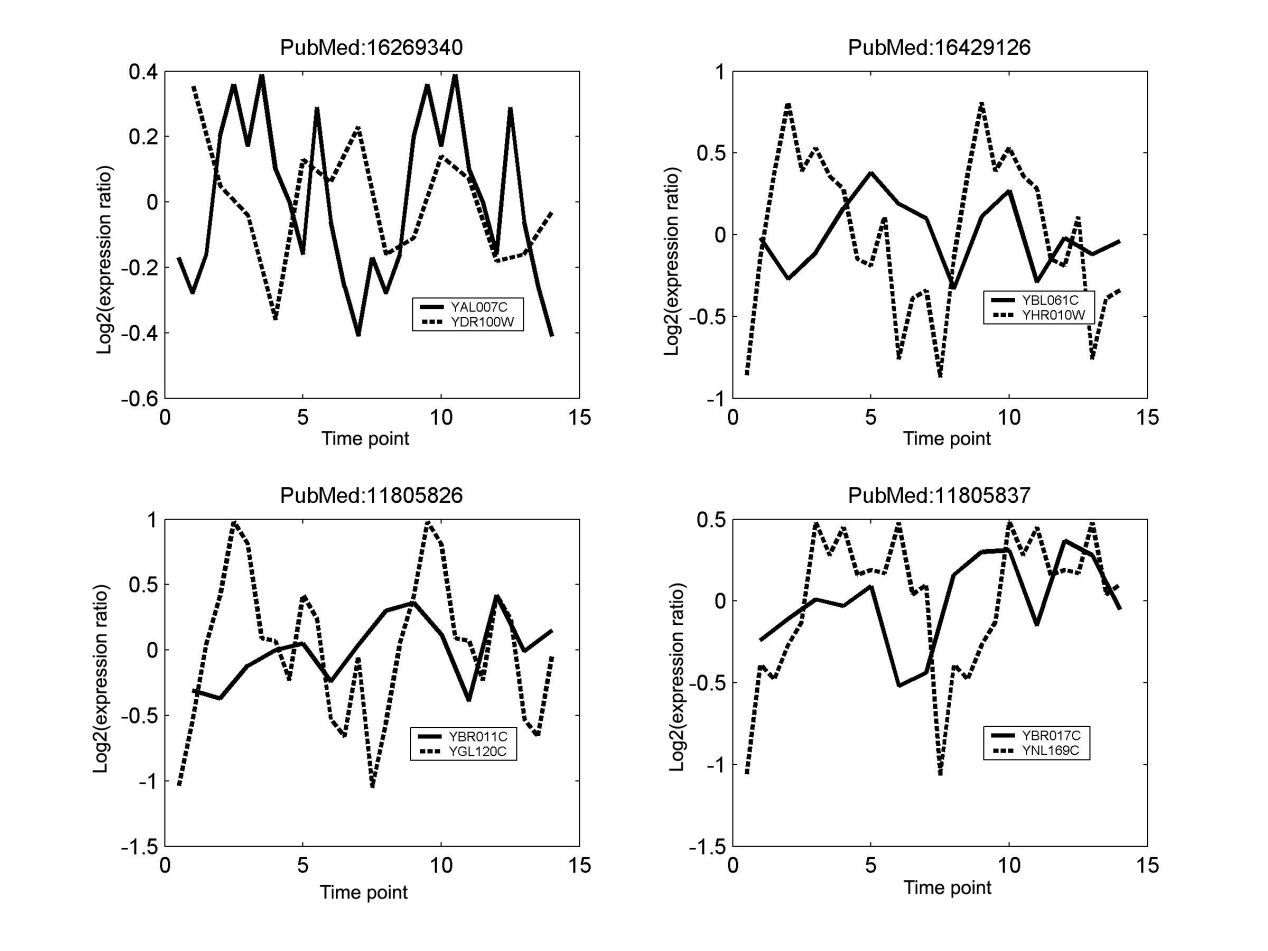
**Examples of BioGRID pairs that are 1:2 or 2:1 phase locked in their expression time series**. The frequency of the slower time series has been doubled. The PubMed ID gives the literature that contains evidence of their interaction. Data from the ELU experiment.

The basis of higher order phase locking is the different intrinsic dynamic time scales of the different time series. Figure 9 presents some examples of order 2 locked BioGRID gene pairs. The corresponding PubMed ID that contains evidence of their interaction is also given. More examples of high order locked pairs from the cell cycle genes, and TF-target pairs are given in Additional files 4 &5. In these examples, the values of *λ*_1,1, _and *r *are low, and interaction would have been missed if using them as the metric. Demo movies of higher order phase locking are available at our website http://zen.dom.uab.edu:8080/phase/demo/. The wide range of phase lags and the significant proportion of higher order locking emphasize the need of a rigorous method like the phase locking analysis that can automatically take care of them.

#### Agreement between the 4 datasets

**Figure 10:**
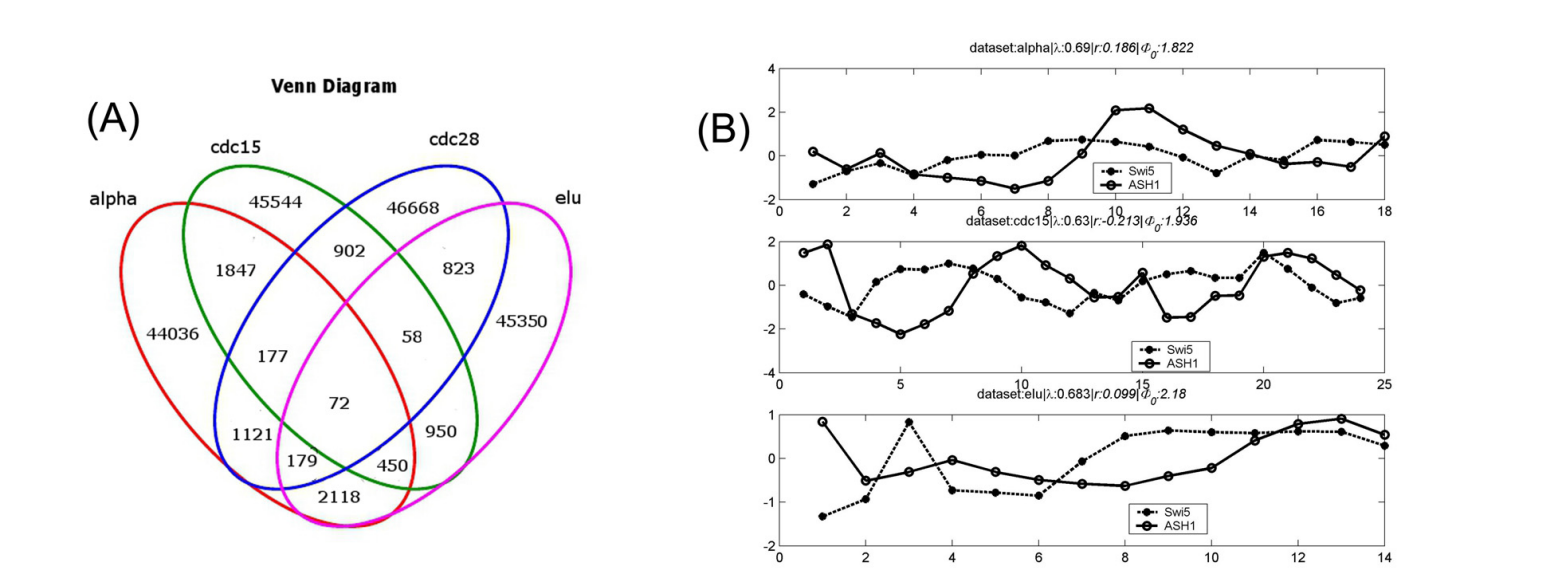
**Agreement among the 4 datasets**. A. Venn diagram of sharing of the phase locked pairs in the four datasets. (B) A TF-target pair that shows phase locking across three datasets, but exhibits low correlation consistently.

As observed by others, we see large variation among the 4 datasets. However, when we examine the phase locked pairs, we found that there is significant concordance between the four datasets, with p < 0.0014 (Fisher's exact test). The Venn diagram is given in Figure 10A. Interestingly, we find that often for gene pairs that show consistent phase locking in multiple datasets, their correlation do not follow the trend. Figure 10B is an example, where the TF-target pair Swi5-ASH1 [42], shows high *λ *consistently in the alpha, cdc15 and ELU experiments, but their expressions correlate in no dataset.

### Network inference using phase locking

In our study so far, we have demonstrated that phase locking in expression changes is a good indicator of interaction. It is therefore natural to utilize it to construct gene interaction networks.

#### Highly connected genes tend to be essential genes

**Figure 11:**
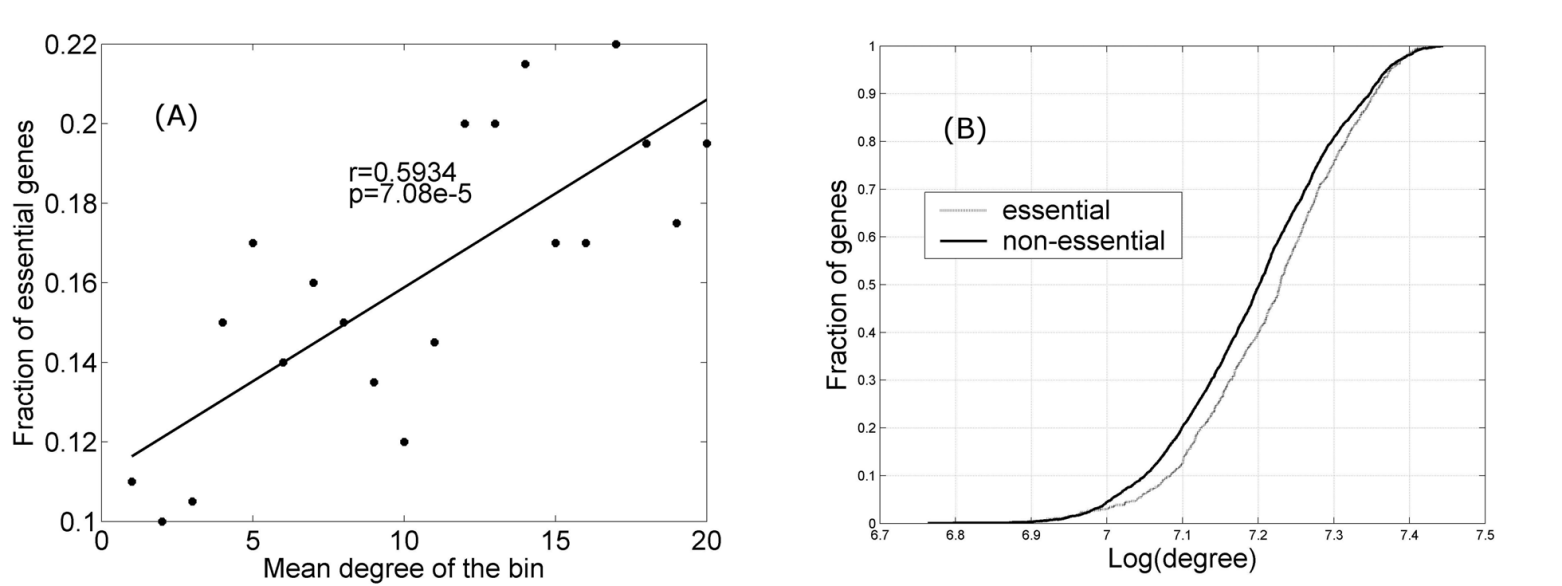
**Genes of higher network degree are more likely to be essential genes**. (A). Fraction of essential genes goes up linearly with increasing network degree (*r *= 0.59, p ~ 7e-5). (B) Cumulative distribution fraction (CDF) plot shows that the network degree distribution of essential genes is skewed toward having higher network degrees (p < 1e-5, KS test).

We first examined the network defined by the adjacency matrix given in equation 9. Analysis of the yeast protein-protein interaction network in the past has revealed that highly connected genes are more likely to be essential for survival [43]. In a recent study of co-expression network by Zhang *et al *[44], strong positive correlations between the network degree and the functional essentiality of genes were also observed. In our phase locked networks, we also observed a positive correlation between the network degree *k_i _*of a gene and its likelihood of being essential. The relationship is nicely captured In Figure 11A. All genes were ranked according to their *k_i _*and partitioned into 20 equal sized bins. The proportion of essential genes in each bin was then plotted against the mean *k_i_*. A linear dependence is evident (*r *~ 0.6, p ~ 7e-5). We also examined the degree distribution of essential versus non-essential genes, given in Figure 11B. Compared to the non-essential ones, essential genes are significantly skewed toward having higher numbers of phase locked partners (p < 1e-5, KS test).

#### Genes in network modules have focused biological themes

**Figure 12:**
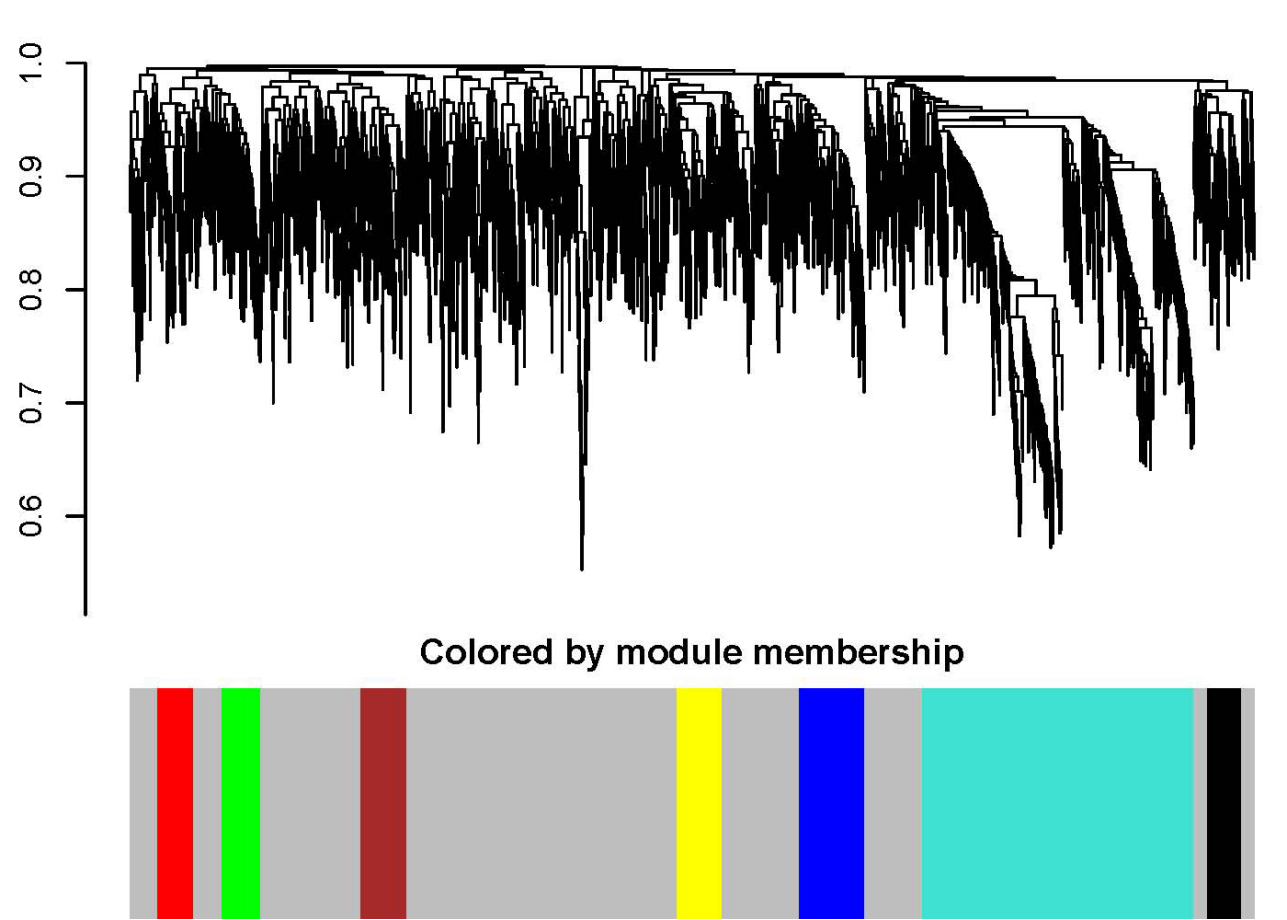
**Network modules identified based on phase locking in the alpha factor dataset**.

The structure of the phase locked network is further studied through hierarchical clustering using the topological overlapping matrix given in equation 10. As depicted in Figure 12 modular organization is evident. Genes in the grey regions did not belong to any modules (which we call the scattered set). Most of the modules (five out of seven) contain higher numbers of essential genes than the scattered set (p < 4.9e-05, χ^2^-test). Based on the theory of phase locking and our results so far, one anticipates genes from the same module to be highly interactive among themselves. This is indeed true for all the modules when we annotate them with BioGRID information (p < 0.02). In addition, ontological analysis of each identified module was performed using GOstat [45], to examine the biological themes and the functional relationship of the module members. We found that genes in the same module tend to be involved in a set of focused signaling pathways. At a stringent cutoff p = 0.01, genes in the 7 modules share a total of 65 GO categories, and 105 at p = 0.05, whilst random gene sets of the same sizes and the scattered gene set do not share any GO category even at a very loose cut off p = 0.10. In Table 1 we list the GO Biological Processes shared by genes in each module at p < 0.01. A common theme is evident that most of these processes are critical to DNA replication and cell cycle regulation. For instance, genes in the turquoise module likely form a cell cycle regulatory module, while the yellow module is likely associated with RNA processing.

**Table 1:** Top GO Biological Processes shared by the genes in the 7 modules shown in Figure 12, at p < 0.01.

GO Accession #	Description	Module
GO:0006412	translation	Red

GO:0044249	cellular biosynthetic process	Red

GO:0009059	macromolecule biosynthetic process	Red

GO:0042254	ribosome biogenesis	Green

GO:0022613	ribonucleoprotein complex biogenesis	Green

GO:0006412	translation	Brown

GO:0009059	macromolecule biosynthetic process	Brown

GO:0044249	cellular biosynthetic process	Brown

GO:0044267	cellular protein metabolic process	Brown

GO:0019538	protein metabolic process	Brown

GO:0044260	cellular macromolecule metabolic process	Brown

GO:0009058	biosynthetic process	Brown

GO:0010467	gene expression	Brown

GO:0006364	rRNA processing	Yellow

GO:0016072	rRNA metabolic process	Yellow

GO:0042254	ribosome biogenesis	Yellow

GO:0006396	RNA processing	Yellow

GO:0022613	ribonucleoprotein complex biogenesis	Yellow

GO:0016070	RNA metabolic process	Yellow

GO:0010467	gene expression	Yellow

GO:0006139	nucleobase, nucleoside, nucleotide and nucleic acid metabolic process	Yellow

GO:0022613	ribonucleoprotein complex biogenesis	Turquoise

GO:0042254	ribosome biogenesis	Turquoise

GO:0000278	mitotic cell cycle	Turquoise

GO:0022402	cell cycle process	Turquoise

GO:0007049	cell cycle	Turquoise

GO:0006412	translation	Turquoise

GO:0044249	cellular biosynthetic process	Turquoise

GO:0006396	RNA processing	Turquoise

GO:0022403	cell cycle phase	Turquoise

GO:0006364	rRNA processing	Turquoise

GO:0000074	regulation of cell cycle	Turquoise

GO:0006261	DNA-dependent DNA replication	Turquoise

GO:0006260	DNA replication	Turquoise

GO:0016072	rRNA metabolic process	Turquoise

## Discussion

### The importance of phase space information

Synchrony through oscillation is a common, maybe the most efficient, way to coordinate regulation in complex nonlinear dynamic systems. It is also a ubiquitous phenomenon in biological systems, where oscillations are observed in all organisms across a wide range of temporal and spatial scales, and are believed to play an important role in maintaining homeostasis and delivering encoded information [22,46,47]. Examples include the synchronized oscillations in interneuron networks, pulsatile endocrine hormone secretion, circadian oscillators, Somite segmentation, and innumerable others. Higher order phase locking occurs frequently, reflecting the multi-stability of complex systems, and is believed important to function. For instance, in the study of cardiorespiratory synchronization, when plotting the instantaneous respiratory phase at the occurrence of a heartbeat versus time, Schäfer and co-workers found *n*:1 synchronization between heart and respiration [48]. In a study with anesthetized rats, Stefanovska *et al*. further observed lengthy synchronization epochs, and transitions from one ratio to another. They suggested that such transitions might be useful in monitoring depth of anesthesia [49,50].

Increasing evidence suggests that, as in many other complex systems in nature, oscillation and frequency modulation is also a general strategy for an organism to coordinate multi-gene responses to external signals [22]. It is found that the transcription factor activities, rather than levels of transcription factor expression, mediate transcriptional regulations [26]. In the negative feedback loop between the tumor suppressor p53 and the oncogene Mdm2, p53 is expressed in a series of discrete pulses after DNA damage, leading to oscillations in Mdm2 [24,25]. The amplitude of the oscillations was much more variable than the period, suggesting strong temporal regulation. In the NF-κB signaling pathway, NF-κB (RelA) localization showed asynchronous oscillations following cell stimulation that decreased in frequency with increased IκBα transcription. Transcription of target genes depended on its oscillation persistence, and thus the functional consequences of NF-κB signaling likely depend on temporal characteristics of the oscillations [27]. In yeast cells it has been shown for several calcium stress responsive TFs (Crz1 and Msn2) that calcium concentration controls the frequency, but not the duration, of their oscillatory localization bursts, and the oscillation propagates to the expression of downstream genes. It has been argued that such frequency modulation of localization bursts ensures proportional expression of multiple target genes across a wide dynamic range of expression levels [23].

These facts all imply the importance to study synchrony in expression oscillation, to understand the information encoded and the underlying interaction/regulation mechanisms. Data from these studies [22-27] also indicate that oscillations in gene expression, like most other oscillations in biological systems, are often pulsatile rather than harmonic. Therefore, mathematical methods rooted from phase space analysis are desirable. The latter can potentially lead to new efficient network modeling algorithms, and help to understand the complex spatial-temporal architecture of transcriptome.

### Advantages and limitations of the phase locking analysis

Additional to the theoretical appeals, we believe that phase locking analysis has several advantages in clustering genes of similar patterns and in network modeling. Firstly, compared to approaches that primarily rely on the similarities in the amplitude domain patterns, phase locking utilizes the dynamics underlying the temporal pattern, which is more robust against noise. This is particularly appealing in network modeling of gene expression data, as they usually contain high noise. Also the transcript abundance measurements often reflect a compressed, even altered representation of the true expression changes due to technical issues [51-54]. These can significantly mask the true patterns in amplitude changes. In contrast, phase locking analysis, which focuses on the timing of the changes, will be less affected by the noise and the technical issues in the microarray gene expression study. In fact, it is known that noisy coupled nonlinear dynamic systems may synchronize in phase whilst their amplitudes remain uncorrelated [31,55]. In our analysis, we have seen ample examples where interacting gene pairs (according to BioGRID or ChIP-chip) exhibit obvious phase locking, but have very low correlation (See Figure 9 and Additional files 2, 4 and 5).

Secondly, in phase locking analysis, the phase lags in gene expression changes between different genes are automatically accounted for, and the performance is not affected by the amount of lag. On the other hand, the performance of alignment approaches depends on whether the lag is close to an integer number of the time steps of the experiment, and they need to be adjusted for each pair as the lags of different gene pairs vary greatly.

Thirdly, phase locking does not require the two time series to have the same dynamic time scale, or the same frequency. It is known that some pathways or gene groups in a cell respond to external signals at a much faster time scale than others [11]. High order m:n phase locking analysis can take care of such interacting gene pairs, whilst they would be missed by the alignment method.

A limitation of the phase locking analysis is its reliance on the temporal spectrum to accurately derive the instantaneous phase, which could significantly affect results when either the number of sampling points or the sampling frequency is too low. Note that higher sampling frequencies are needed to detect high order phase locking. We have carried out a set of numerical simulations and observed significant deterioration in performance when the number of sampling points is reduced to ~5 or lower (data not shown). This limitation is not unique to the phase locking analysis. All pair-wise alignment approaches suffer from the same limitations as they all rely on adequate sample size to make a good assessment of whether the expression patterns of the pair are similar or not. There is also a caveat with the application of phase locking analysis to network modeling. Gene interactions or phase locking occurs inside each cell. High throughput time series gene expression studies commonly measure a population of cells all at one time, effectively averaging the expressions of each gene across the whole cell population. In processes where there is high synchrony in the whole cell population, such as the cell cycle study presented here, phase locking between gene expression changes that occur inside each cell is preserved at population level, and can be detected from population measurements. In other biological processes, where cellular heterogeneity plays a key role, information of the signaling dynamics and phase locking inside each cell could be lost in population-level measurement. Again, in such situations, the performance of the co-expression alignment approaches to detect interaction will also be affected.

Lastly, the recent advancements in single-cell techniques has enabled the generation of time-series gene expression measurements in a large number of individual living cells [21]. We believe that phase locking analysis will be particularly suitable for such data. The dynamic information at the level of individual living cells will be critical to unravel how a genetic network operates at the systems level.

## Conclusion

A major challenge in systems biology is to reconstruct gene networks that are involved in basic cellular processes, and to understand how alterations to the network affect functions and consequently phenotypes. Interactions between genes can result in expression amplitude variations as well as temporal patterns. Therefore, network inference utilizing temporal domain information deserves more attention. In this study, we investigated the potential of the phase locking analysis in network modeling of time course gene expression data. We demonstrated that interacting gene pairs, including transcription regulation interaction, protein-protein, or genetic interaction, are more likely to exhibit phase locking in their gene expression changes, and vice versa. Among the phase locked pairs, up to ~10% are contributed from higher order locking, and the relative phase difference spans across the whole range of [-π, π]. Based on these findings, we constructed interaction networks and revealed that genes with higher network degrees are more likely to be essential genes. Utilizing the phase-locking index based topological overlapping matrix, we further investigated the modular structures in the network. We showed that genes forming network modules are more likely to be essential genes than scattered genes in the network, and members of the same module tend to be involved in the same biological functions and processes. In view of the importance of the frequency domain signal in transcription regulation, we believe that the phase locking analysis can potentially lead to new network modeling approaches and help to understand the dynamic designs of the intracellular signaling networks.

## Methods

### Gene expression data

Yeast cell cycle gene expression data were downloaded from the Yeast Cell Cycle project at the Stanford University http://genome-www.stanford.edu/cellcycle/data/rawdata/. These studies profiled expression changes in 6178 genes at ~20 time points under each condition following alpha factor arrest (18 time points from 0-119 minutes), elutriation ELU (14 time points from 0-390 minutes), and arrest of a cdc15 (24 time points from 10-290 minutes) and a cdc28 (28 time points from 0-160 minutes) temperature sensitive mutant [33,34]. Many genes have missing data points. The cdc28 data is the most severely affected, ~80% of genes contains at least 1 missing values. For the other three datasets, it ranged 6-27%. In this study, we kept genes that had at most 1 missing data point in each data set for further analysis. Among all 6178 genes profiled, 144 are annotated by the Gene Ontology (GO, http://www.geneontology.org/) to be involved in the biological process of cell cycle (Since the start of our study, more genes have been annotated to be involved in this biological process. The number is now higher than 144). They are termed cell cycle genes in this study.

### ChIP-chip data of transcription binding

Simon *et al *studied the transcription regulation of yeast genes by 9 cell cycle regulating transcription factors (TF): Fkh1, Fkh2, Ndd1, Mcm1, Ace2, Swi5, Mbp1, Swi4, and Swi6, using the ChIP-chip technology [36]. We have obtained their data and used it as benchmark of transcription interaction. For each TF, the study derived a binding p-value for each gene which reflects the likelihood that the TF binds to the promoter of this gene. We log-transformed the p-value to a significance score by

(1) $$sig=-log_{10}p$$

For each gene *i*, the Z-score of the *sig *across the 9 TFs is also determined to examine binding specificity.

We constructed a positive control target set for each TF that consists of those with *sig *> 3 (significant binding), and the Z-score > 1.5 (the binding is specific). The number of targets for each TF ranges from 18-54 for the alpha factor arrest data set, 12-50 for the cdc15 dataset, 1-21 for the cdc28 dataset, and 19-65 for the ELU data set. A negative control non-target set is constructed for each TF that includes all genes with *sig <*1 (*p *> 0.1). This set consists of over 3,000 genes for each TF in the alpha factor and cdc15 datasets, over 875 for each TF in the cdc28 dataset, and over 4,000 for each TF in the ELU dataset.

### Phase locking analysis

We adopt the analytic signal concept [31] to derive the phase of an arbitrary signal. Briefly, given a time series *s*(*t*), its Hilbert transformation [31,56] is given by

(2) $$s_{H}(t)=\frac{1}{\pi }\;\text{PV}\int_{-\infty }^{\infty }\frac{s(t)}{t-\tau }d\tau$$

where PV stand for Cauchy Principal Value of the integration [57]. The corresponding analytical signal can then be constructed

(3) $$s(t)+is_{H}(t)=A(t)e^{i\varphi (t)}$$

where the instantaneous phase *φ *(*t*) is thus uniquely determined. *φ *(*t*) calculated this way can be sensitive to low-frequency trends [31]. We used Matlab's detrend function to remove low frequency trends in data, and the following string of commands to obtain the instantaneous phase for the time series of each gene *g_j_*(*t*): [31,58].

(4) $$\varphi_{j}(t)=\text{unwrap}(\text{angle}(\text{Hilbert}(\text{detrend}(g_{j}(t))))$$

For two time series with instantaneous phase *φ_i_*(*t*) and *φ_j_*(*t*), their cyclic relative phase is determined by

$$\Psi (\text{t})=(\varphi_{i}(t)-\varphi_{j}(t))\;\text{mod}(2\pi )$$

If two time series interact with each other resulting in phase locking, Ψ = Ψ_0 _is a constant. On a Poincare phase map this will be represented by a stable fixed point. For noisy time series the phase difference is in general not a constant but distributes around Ψ_0 _: |Ψ - Ψ_0 _| <*const *, where Ψ_0 _is the average relative phase shift, and the significance of phase locking can be assessed in the statistical sense [31]. The phase locking can be a general (*m*:*n *) locking, with

(5) $$\Psi_{\text{m},\text{n}}(\text{t})=(m\varphi_{i}(t)-n\varphi_{j}(t))\;\text{mod}(2\pi )$$

constrained around a constant value Ψ_0_, where *m *and *n *are integers.

To evaluate the significance of phase locking, we utilize the circular mean of the phase difference

(6) λm,n=|〈exp(iΨm,n(t))〉|=|((1tN)∑l=1Nexp(iΨm,n(tl)))|

In a perfect locking *λ *_*m*, *n *_= |exp(*i*Ψ_0_)| = 1, and *λ *_*m*, *n *_→ 0 when Ψ_m_,_n_(*t*) is randomly distributed.

The mean relative phase Ψ_0 _is calculated by

(7) $$\begin{array}{l} \Psi_{0}=\arg\left(\langle \exp\left(i\Psi_{\text{m},\text{n}}(t)\right)\rangle \right)\\ \;\;\;\;\;\;\;\;=\arg\left((\frac{1}{t_{N}})\sum_{l=1}^{N}\exp\left(i\Psi_{\text{m},\text{n}}(t_{l})\right)\right) \end{array}$$

where arg is a mathematical function operating on complex numbers and gives the angle. Note that the value of λ_m, n _is not affected by the value of the relative phase difference Ψ_0 _; the two time series can have any amount of phase lag.

In this study, we considered locking with Max{*m, n*} up to 4, and define the final phase locking index to be

(8) $$\lambda =\max\{\lambda_{m,n},m,n=1,2,3,4\}$$

Here max is used because the two genes are considered phase locked if the value of anyone of the *λ *_*m, n*_'s, is high. We do not think there is a need to consider higher order than 4 due to the limited number of time points in these datasets, and the noise in microarray data. In addition, higher order locking is less common and probably unstable in the presence of noise [59]. We have also investigated several other measures of phase locking, including the Shannon's entropy and the intensity of the first Fourier mode of the distribution of Ψ_m, n_, [31]. No significant difference in predictions was found. Therefore, in this study we will only report the results of λ.

### Network modeling

To construct interaction networks, we define the phase locking adjacency matrix (*A_i, j_*) by

(9) $$A_{ij}=\{\begin{array}{cc} 1, & \lambda_{ij}\geq \lambda_{0}\\ 0, & \lambda_{ij}<\lambda_{0} \end{array}$$

Where *λ *_0 _is a threshold. The network degree of each gene *i *thus can be calculated by *k_i _*= ∑*_j _A_ij_*. In this study, *λ *_0 _is chosen to be *μ *+ 2*σ *(*i.e*. Z-score = 2), where *μ *and *σ *are the mean standard deviation of *λ *between random gene pairs. Namely, gene pairs with the value of their phase locking index at least two standard deviations above mean of all pairs are considered significantly phase locked. In the 4 yeast cell cycle data sets, *λ *_0 _~0.75 - 0.80. When we compare phase locking networks with the networks predicted using the correlation coefficient *r*, a same *Z *= 2 cutoff was used.

The topological overlapping matrix is defined following the same strategy as in [60], by

(10) $$T_{ij}=\{\begin{array}{cc} \frac{\sum_{l}\lambda_{il}\lambda_{jl}+\lambda_{ij}}{\min\{n_{i},n_{j}\}+1-\lambda_{ij},}, & i\neq j\\ 1, & i=j \end{array}$$

where *n_i _*= ∑*_j _λ _ij _*is the node connectivity. *T *measures the sharing of first degree phase locked neighbors, and is designed to identify the modular structure in interaction networks. Hierarchical clustering is then performed using *T_ij _*as the similarity measure to identify the network modules.

## List of abbreviations used

AUC: area under curve; CDF: Cumulative Distribution Fraction; GO: Gene Ontology; KS test: Kolmogorov-Smirnov test; OR: odds ratio; ROC: receiver operating characteristic; TF: transcription factor.

## Authors' contributions

SG, JH, MJH and XW designed the study. SG, XW and JC wrote the algorithms and performed the analysis. SG and XW wrote the manuscript and created the figures and tables. All authors read and approved the final version of the manuscript.

## Supplementary Material

Additional file 1 **TF-target pairs with high phase locking index**. Click here for file

Additional file 2 **Phase lagged BioGRID examples**. Examples of BioGRID pairs that are phase locked in their expression time series, but with low time-lagged correlation. The maxim absolute values of lagged correlation are calculated with time lags from -4 to 4. This clearly shows that time-lagged correlation failed to capture the association when the time lag is not an integral number of sampling step in the experiment. Click here for file

Additional file 3 **Number of *m*:*n *phased locked cell cycle gene pairs (TF-target pairs) at different values of *m *and *n***. Click here for file

Additional file 4 **Examples of 1:2 lock phase locked cell cycle gene pairs**. The top 16, max{m, n} = 2, phase locked cell cycle gene pairs from the cdc15 arrest dataset. (A) When the original expression profiles of the locked pairs are presented, phase locking is not immediately visible. (B). When the frequency of one partner is doubled, phase locking is evident. All pairs have low *λ *_1,1_(< 0.13,) or *r*(< 0.35). Click here for file

Additional file 5 **Examples of 1:2 phase locked TF-target pairs**. Expression profiles of the top five 2:1 phase locked Swi5-target pairs in the cdc28 dataset. Top panel: original expression profiles. Dashed line: Swi5; Solid line: target genes; Bottom panel: The frequency of the Swi5 profile has been doubled. For all pairs, *λ *_2,1 _> 0.77, *λ *_1,1 _< 0.2, and *r *< 0.45. Click here for file
