# Supplementary figures and images for: Global analysis of phase locking in gene expression during cell cycle: the potential in network modeling
Source: BMC Syst Biol. 2010 Dec 3;4:167. doi: 10.1186/1752-0509-4-167 (PMC3017040; doi:10.1186/1752-0509-4-167)

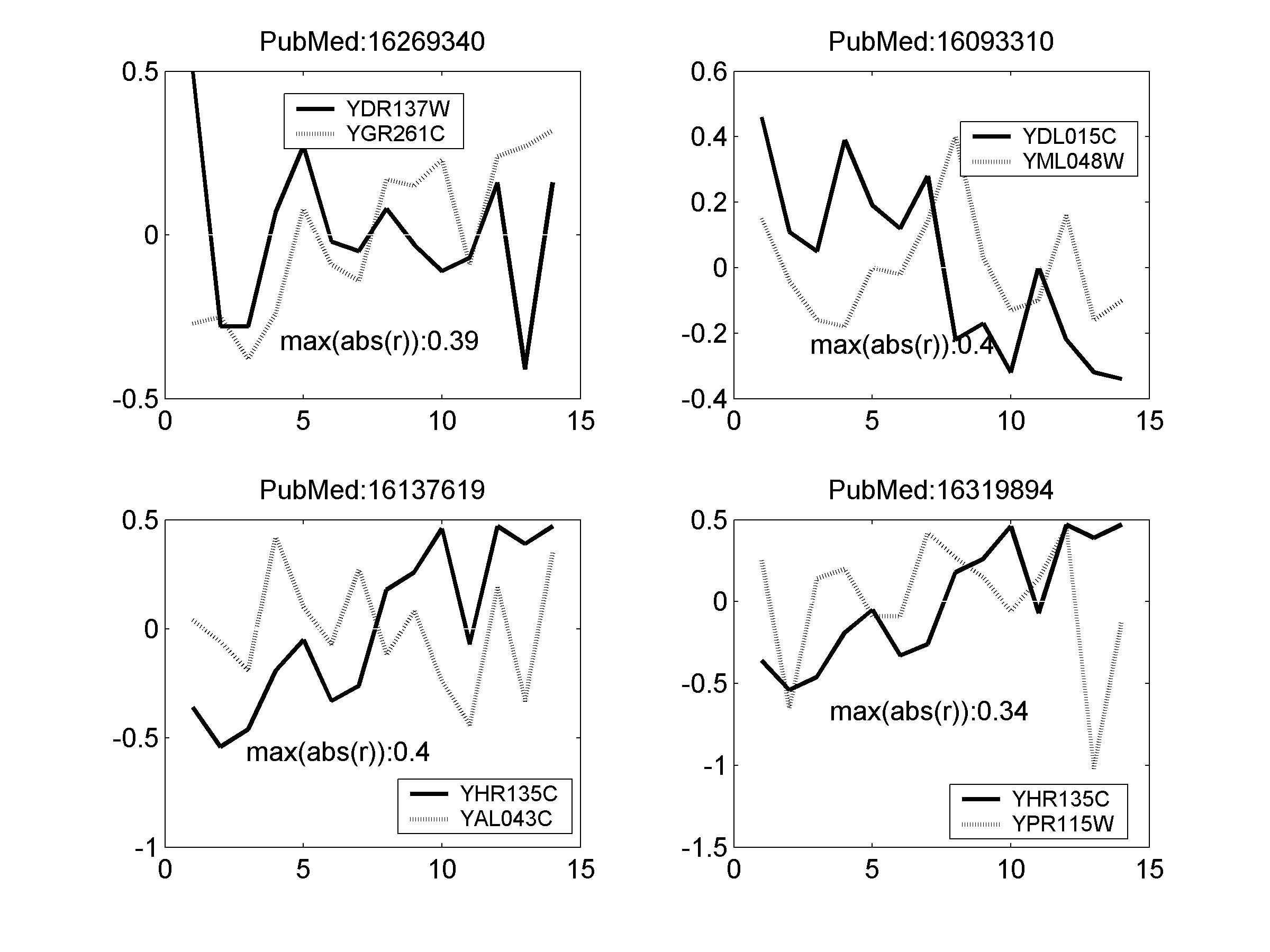

Supplement: Additional file 2 — Phase lagged BioGRID examples. Examples of BioGRID pairs that are phase locked in their expression time series, but with low time-lagged correlation. The maxim absolute values of lagged correlation are calculated with time lags from -4 to 4. This clearly shows that time-lagged correlation failed to capture the association when the time lag is not an integral number of sampling step in the experiment. [file 1752-0509-4-167-S2.JPEG]

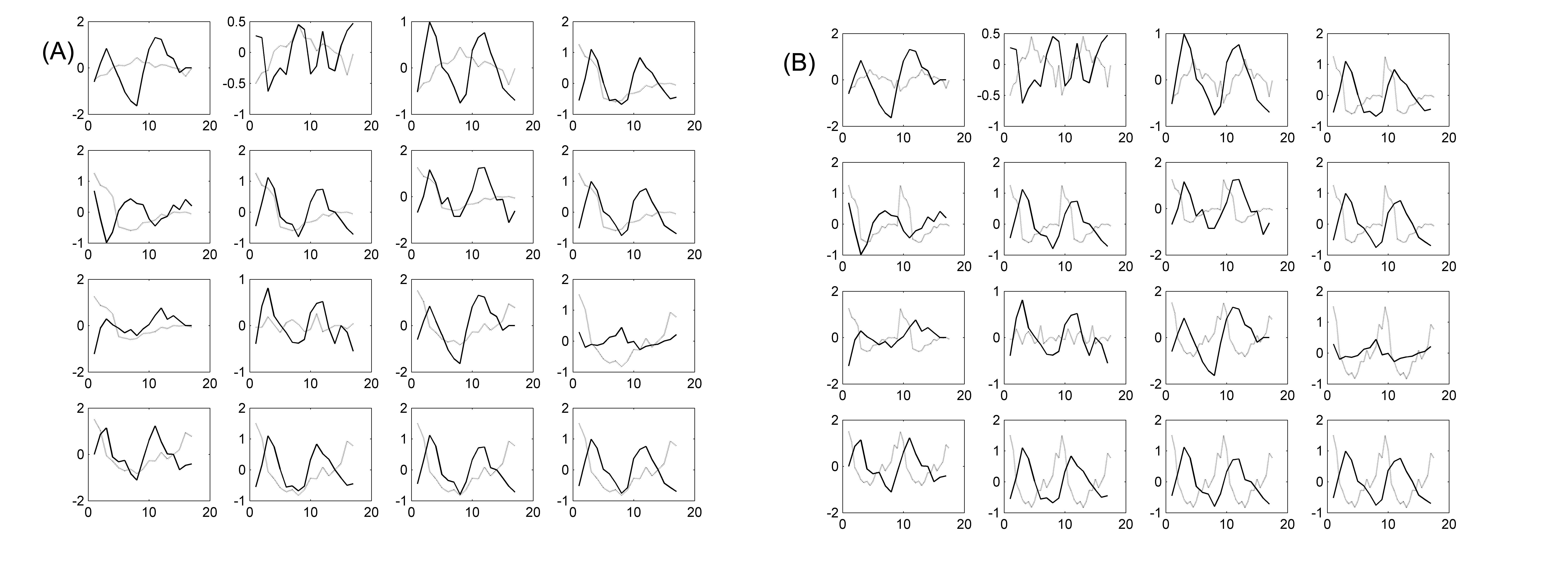

Supplement: Additional file 4 — Examples of 1:2 lock phase locked cell cycle gene pairs. The top 16, max{m, n} = 2, phase locked cell cycle gene pairs from the cdc15 arrest dataset. (A) When the original expression profiles of the locked pairs are presented, phase locking is not immediately visible. (B). When the frequency of one partner is doubled, phase locking is evident. All pairs have low λ 1,1(< 0.13,) or r(< 0.35). [file 1752-0509-4-167-S4.JPEG]

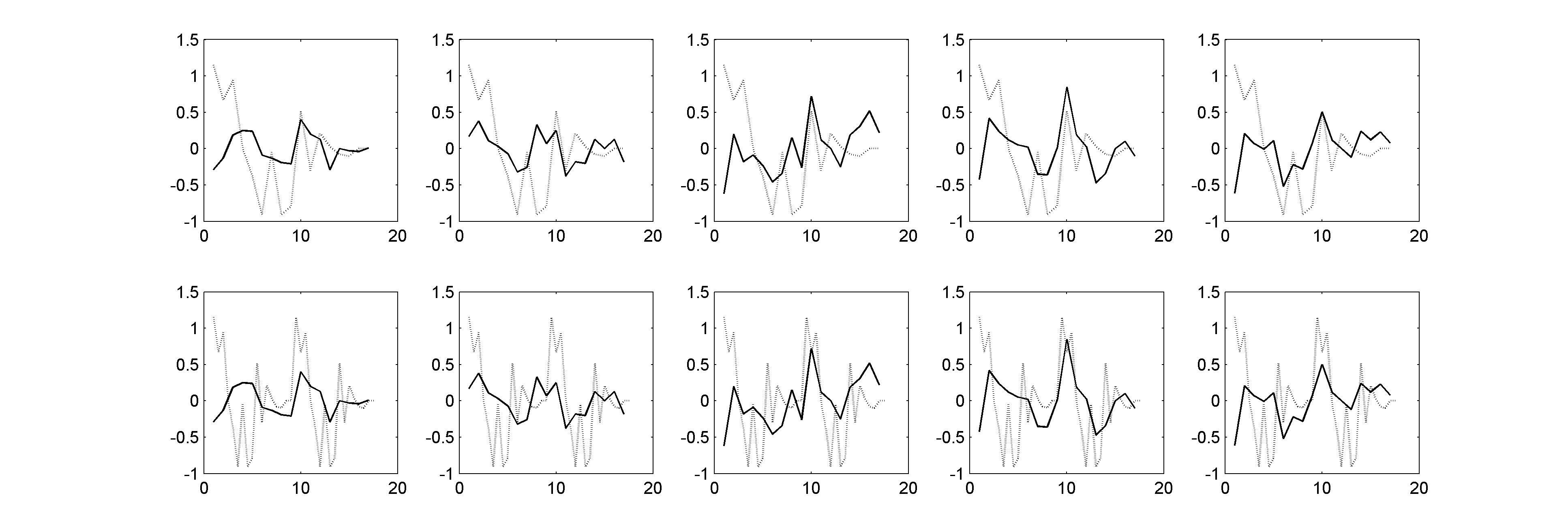

Supplement: Additional file 5 — Examples of 1:2 phase locked TF-target pairs. Expression profiles of the top five 2:1 phase locked Swi5-target pairs in the cdc28 dataset. Top panel: original expression profiles. Dashed line: Swi5; Solid line: target genes; Bottom panel: The frequency of the Swi5 profile has been doubled. For all pairs, λ 2,1 > 0.77, λ 1,1 < 0.2, and r < 0.45. [file 1752-0509-4-167-S5.JPEG]
