# Supplementary material for: Global analysis of phase locking in gene expression during cell cycle: the potential in network modeling
Source: BMC Syst Biol. 2010 Dec 3;4:167. doi: 10.1186/1752-0509-4-167 (PMC3017040; doi:10.1186/1752-0509-4-167)
Supplement: Additional file 3 — Number of m:n phased locked cell cycle gene pairs (TF-target pairs) at different values of m and n. [file 1752-0509-4-167-S3.DOCX]

| (m, n) | (1,1) | (1,2) | (2,1) | (1,3) | (3,1) | (1,4) | (4,1) | (2,3) | (3,2) |
| --- | --- | --- | --- | --- | --- | --- | --- | --- | --- |
| Dataset |  |  |  |  |  |  |  |  |  |
| alpha | 641(1030) | 12(21) | 9(19) | 8(1) | 4(3) | 9(6) | 26(2) | 6(5) | 5(6) |
| cdc15 | 550(908) | 5(0) | 3(2) | 1(0) | 2(0) | 1(0) | 2(0) | 1(0) | 1(0) |
| cdc28 | 807(2252) | 5(31) | 8(109) | 0(2) | 0(8) | 1(7) | 3(0) | 4(8) | 4(20) |
| ELU | 311(2085) | 9(218) | 6(96) | 1 (13) | 0(9) | 1(33) | 1(3) | 2(55) | 1(37) |
